# Editorial

**Published:** 1863-02

**Authors:** 


### Editorial.

### MEDICAL DEPARTMENT OF LIND UNIVERSITY.

The delay in issuing the *Examiner* enables us to notice, briefly, the closing exercises of the fourth annual course of instruction in this Institution. The public commencement was held on the evening of the first Tuesday in March, 1863, in the hall of the College. The room was well filled. The President of the Faculty, Prof. H. A. JOHNSON, conferred the degree of M.D. upon seventeen members of the class, and gave them a short, but eloquent and impressive charge in relation to their duties and responsibilities. The exercises were closed with an interesting valedictory address, which will be found in the present number of the *Examiner*.

At the close of the public exercises, the graduates, students, and alumni of the College, together with the members of the Chicago Medical Society, repaired to the residence of Prof. N. S. DAVIS, where an elegant social entertainment was enjoyed until a late hour.

The number of matriculants during the past term was 81, and the number of graduates 17.

The next collegiate year commences, with a regular clinical and reading term, on the *first* Monday in April, and continues until the first Monday in August. During this period, the class in attendance will have a regular clinical lecture in the medical wards of the Mercy Hospital every Monday, Tuesday, and Thursday mornings, at 9 o'clock, by Prof. N. S. DAVIS; a surgical clinic in the surgical wards of the Mercy Hospital every Friday morning, and another at the College Dispensary every Wednesday afternoon, by Prof. E. ANDREWS; a medical clinic at the College Dispensary every Tuesday afternoon, by Prof. H. WING; and a clinic on diseases of women and children at the same place every Saturday afternoon, by Prof. W. H. BYFORD. Besides these ample clinical advantages, the course embraces one examination and familiar explanatory lecture on some one of the branches of medical study every day. This well-arranged clinical and reading term, added to the full annual lecture term of five months, and the systematic arrangement of the field of study into junior and senior branches, makes up a system of collegiate instruction, in all the departments, equal to that of the best schools, either in this country or Europe.

---

### ILLINOIS STATE MEDICAL SOCIETY.

We trust that none of our readers will forget that the regular Annual Meeting of the State Society is to be held in Jacksonville, on the first Tuesday in May. So many of the members are in the Army, that it becomes more necessary for those who remain at home to be punctual in maintaining all our social and professional organizations.

Let none stay away through fear that the meeting will be a failure, for it will certainly be held, and will be attended by a sufficient number of members to render it pleasant and profitable. The opportunity to visit the State Institutions for the Insane, Deaf and Dumb, and Blind, will also add much to the interest of the occasion. We expect to meet a good representation from all parts of the state.

### CHICAGO MEDICAL SOCIETY.

At the regular Annual Meeting of the Chicago Medical Society, held April 3d, 1863, the following officers were chosen for the ensuing year:—

*For President.*—GERHARD PAOLI, M.D.

*Vice-President.*—M. O. HEYDOCK, M.D.

*Secretary and Treasurer.*—E. L. HOLMES, M.D.

*Delegates to the Illinois State Medical Society.*—Drs. E. L. HOLMES, D. D. WAITE, ORREN SMITH, and HIRAM WANZER.

*Delegates to the American Medical Association.*—Drs. IRA HATCH, A. FISHER, G. PAOLI, and J. M. TUCKER.

*Sanitary Committee.*—A. FISHER, E. L. HOLMES, and HIRAM WANZER.

During the past year, the Society has maintained an active and prosperous existence,—profitable to its members and honorable to the profession.

---

AMERICAN MEDICAL ASSOCIATION.—We are happy to assure our readers, that the call for a Meeting of the Association, on the first Tuesday in June, is cordially endorsed by the Medical Journals, and the Profession, in all parts of the country. The letters we receive, and the reports of the appointment of Delegates from various sections, indicate a full and profitable meeting. We hope the Profession in our own State, and throughout the North-West, will take prompt measures to ensure a full representation.

Let the local societies, whether city, county, or district, whose meetings may have been neglected, during the two preceding years of national excitement, be immediately called together, and Delegates appointed who will be sure to attend the Great Annual gathering of our brethren. In counties or districts, in which no social organizations exist, let the practitioner procure a letter of recommendation from some permanent member of the National Association, and present himself at the meeting, and he will be admitted as a member by invitation. Hence there is no excuse for any to remain at home. We would also urge those who are on Committees, either standing or special, to make ready their reports, and if any have voluntary papers or communications to present, let them place a copy in the hands of the Committee of Arrangements early, that they may be refered to the proper sections in time for fair consideration. Let no one, outside of Chicago, imagine that the course taken by the *Chicago Medical Journal*, and its senior editor, Professor D. BRAINARD, in opposing the meeting of the Association, indicates any division of sentiment or action in the Profession here, or that it represents the wishes or feelings of any one here but himself. On the contrary, the Profession here are united, and earnestly preparing to give their brethren as cordial and pleasant a reception as they have met with in any other city in our country. Our hotels are of the best character, and amply sufficient to accommodate half a dozen "Canal Conventions" and Medical Associations at the same time. We have full confidence that the coming annual meeting will be well attended; that its members will transact the legitimate business of the Association with dignity, harmony, and profit; that they will revive and extend past associations and friendships, and by their liberality of sentiment, and their strict adherence to the proper objects of the Association, they will set an example worthy of imitation by all other conventional organizations, whether religious, political, or scientific.

---

THE NEW YORK OPHTHALMIC SCHOOL AND HOSPITAL held its Eleventh Anniversary on the 24th inst., in the University Medical College in Fourteenth Street, before a large and highly intelligent audience. The exercises of the evening were introduced with prayer by the Rev. EDWARD THOMSON, M.D., Editor of *The Christian Advocate and Journal.* After some introductory remarks by the President, SOLOMON JENNER, Esq., the names of the graduating class were read by Dr. MARK STEPHENSON, lecturer on anatomy, pathology, and treatment of diseases of the eye, who remarked to the President: that thsee young gentlemen were students from the different medical colleges in this city, that they have attended his lectures on ophthalmic surgery, been examined every Saturday by Dr. M.

P. Stephenson, attended the cliniques three days in the week, learned to diagnose diseases of the eye, watched the effect of remedies from day to day, and witnessed the various operations at the hospital, where over 1,000 patients are treated every year; and further said: that he believed they were better qualified to practice this department of their profession than many physicians who have been 20 or 30 years in practice. The diplomas were then presented by Solomon Jenner, President of the Institution, as follows:—A. E. Jenner, M.D., Ohio; J. M. Waddle, Yates County, N. Y.; J. P. Schenck, Junior, Dutchess County, N. Y.; De Witt Webb, Dutchess County, N. Y.; J. H. McCann, M.D., Louisville, Ky.; Robert King, Geneva, N. Y.; H. G. Olmsted, M.D., N. Y. City; James Hutchinson, St. John's, N. B.; Charles P. Sanderson, M.D., Ohio; R. J. Mordon, M.D., Canada West; J. H. Chittenden, Binghamton, N. Y.; Thomas Thompson, Delaware County, N. Y.; G. A. Hayunga, M.D., Canada West; J. H. Hunter, M.D., Concord, N. H.; M. C. Rowland, M.D., Washington County, N. Y.; W. J. Orton, Broome County, N. Y.

The graduates were then addressed in a very forcible and appropriate manner by Dr. Marcus P. Stephenson, one of the attending-surgeons, under whose immediate instruction and examination they had been during the past winter. The Valedictory Address was delivered in a very able and pleasing manner by Alexander E. Jenner, M.D., one of the graduating class; and the exercises of the meeting were closed with an eloquent address by J. P. Garrish, M.D.

---

College Appointments.—It is known to most of our readers that Dr. M. K. Taylor, who occupied the chair of Pathology and Public Hygiene in the Medical Department of Lind University, has been absent, on duty in the Army during all the past two years, and his place in the college has been temporarily filled by others.

He entered the United States service a surgeon to the 26th Regiment of Illinois Volunteers, subsequently, he was promoted to the rank of brigade or staff-surgeon, and is now in charge of the military hospitals at Keokuk, Iowa. The continuance of the rebellion, and, consequently, the necessity of still further absence, caused Dr. TAYLOR to resign his position in the college.

Dr. HENRY WING has been appointed to fill the vacancy. Dr. WING filled the chair, temporarily, during the past winter term, and his permanent appointment will be gratifying to all the friends of the institution.

---

BOOKS AND PAMPHLETS RECEIVED.—We have received a copy of Prof. H. H. Smith's elegant new work on surgery, from the publishers; also, the transactions of the Ohio State Medical Society; transactions of the New York Academy of Medicine; and several reports of asylums and hospitals for the insane, &c., which we have neither time nor space to notice in the present number, but which will be duly attended to in our next issue.

NEW EXCHANGES.—*Canada Lancet:* WILLIAM EDWARD BOWMAN, M.D., Editor, Montreal, Canada; a neatly printed monthly sheet of eight pages.

*The People's Dental Journal:* edited by W. W. ALLPORT, D.D.S., and S. T. CREIGHTON, No. 32 Washington Street, Chicago. This is a neatly printed journal of twenty-four pages, issued quarterly, and well filled with matter of interest to all classes of people.

We cheerfully place both the above on the exchange list.

---

### ACKNOWLEDGMENT.

MESSRS. EDITORS:—I beg leave, through your pages, to acknowledge my indebtedness to Dr. W. B. WITT, Assistant-Surgeon of the 69th Indiana Infantry, for his invaluable services, in thoroughly preparing for my article on the Surgery of the Battles near Vicksburg, a record of the condition of the patients while on the hospital-boats. Dr. WITT is a gentleman of the best attainments, and is an honor to the service.

E. ANDREWS.

TRIBUTE TO ARMY SURGEONS.—It is with pleasure that we copy the following remarks from the *Inquirer*, an able daily newspaper of this City. The tribute is a worthy one, and handsomely bestowed.

Some medical attendants have not been duly qualified, but their number has been greatly exaggerated; and the Surgeons and Assistant-Surgeons of our Army have in their number a body of as devoted men as could be found in the ranks of any service in the world. Testimonies to this effect are multiplied daily in our correspondence. The sufferings of our medical men during the damp, soaking, rainy, season, have been indescribable. Without comfort themselves, in wretched huts in which a farmer could not confine his pigs, lying on the earth or raised from it a few inches by branches of trees, so hard and rough that sleep is almost impossible, the wearied Surgeon may drop off into a slumber; and when the senses are for a few minutes sealed in repose, the exhausted sufferer will often find himself covered with snow, and chilled to the marrow when he awakes to the reality of his situation.

Among his patients, his ingenuity is taxed to the utmost in order to save life, when men are seized with measles and eruptive diseases. Think of thirty or forty men in a regiment, and there are often more, seized with measles, and lying in a damp tent, and rolled up in a soaking blanket, while the dripping clouds keep everything around in utter wretchedness. What can doctors do in such a situation? We have heard of the most amazing displays of self-sacrificing fortitude and devotion to the cause of humanity, by our medical men, in such situations as we have here suggested. And this, too, in the case of men who were reared as tenderly, and nurtured as affectionately as youth could be, in comfortable homes, to which they might still return, but for their devotion to the public service. Sick themselves, and with the prospect of diseased bodies and broken-down constitutions before them, if they should eventually be spared, they hold on their way; and thus they illustrate the extraordinary tendency that exists in the medical mind to deny self for the good of others. Our readers have heard much of the heroism that leads men to the cannon's mouth, but they have heard little of that patient valor, that nobility of intellect, that mastery over the passions, and that self-denial, which our non-combatants are displaying in the steaming atmosphere of the hospital tent, amid the effluvia of typhoid fever, small-pox, measles, and all the diseases that decimate our armies. Treble the amount of salary which the country has awarded to the medical men who are exposing their lives in the field, would not remunerate them for what they do and bear.—*Medical and Surgical Reporter.*

---

McLean Co. Medical Society.—The ninth annual meeting of the McLean County Medical Society met in Dr. A. H. Luce's office, on Monday, April 13, at 1 P.M. Meeting called to order by the President, Dr. Worrell.

Members present, Worrell, Luce, S. Noble Dooly, Ballard, Holderness, Parkhurst, and Park.

On motion of Dr. Luce, Dr. Holderness was elected member of the Society.

The election of officers for the ensuing year being next in order, it was moved, by Dr. S. Noble, that the old officers be continued another year. Adopted.

On motion, the following resolution was unanimously adopted.

*Resolved*, That a committee of three be appointed to wait on Dr. D. L. Crist and inquire into the facts regarding a case of accouchment in which he is reported to have met in consultation with two "Irregular Doctors" of this city, when the grossest ignorance was manifested.

President appointed Drs. H. Noble, C. R. Parke, and A. Elder, the committee to report at next meeting.

Dr. Parke read a paper on the treatment of Hospital Gangrene by Creosote, for which, on motion of Dr. S. Noble, a vote of thanks was tendered, and a copy requested for publication,

On motion, the following gentlemen were selected as delegates to represent the Society in the State Medical Convention at Jacksonville, on the 1st Tuesday in May: Drs. Crothers, Elder, and Ballard.

On motion, Drs. Parke, Fisher, and Worrell, were appointed delegates to the National Medical Convention, which meets in Chicago, on the 1st Tuesday in June, 1863. Alternates, Drs. Parkhurst, Holderness, and Hoover. Essayists, Drs. Cruthers, and Hoover.

On motion, adjourned to meet in Bloomington, on the 2d Monday in July.

Dr. Worrel, *President.*

C. R. Parke, *Sec'y.*

---

Logwood as a Deodorizing Agent.—The *American Medical Review* testifies to the value of logwood as a deodorizer for medical purposes. In cancerous diseases of the uterus, in the form of an injection, it is said to be employed with complete success.—*London Lancet.*

GALVANISM APPLIED, BY AID OF THE LARYNGOSCOPE, TO THE VOCAL CORDS.—The electric current has been brought to bear directly on the vocal cords by Dr. Morell Mackenzie, and two cases of functional aphonia have yielded to it immediately. One patient had completely lost her voice for two years, and had been in London Hospital for some months, where every remedy had been used in vain by Dr. Mackenzie. Cauterization of the larynx, blisters, and even the employment of galvanism externally, had all failed, but the application of galvanism directly to the vocal cords succeeded at once, and, after a week, the patient spoke as well as she had two years previously.

In the other case, where the loss of voice was of eighteen months' duration, and where every kind of treatment had been tried unsuccessfully in the London Hospital, the voice was immediately restored by galvanism directly applied to the vocal cords. Dr. Mackenzie has invented an instrument, by which the electric current can be set going, but does not pass beyond a certain distance till the point is introduced into the larynx, when a spring is touched, and the current reaches the vocal cords. Dr. Mackenzie recommends the remedy in the early stages of clergymen's sore-throat, before the perverted state of the nerves has led to follicular deposit.—*Medical Times and Gazette*, Feb. 7, 1863.

---

RESIGNATION OF PROFESSOR SAMUEL JACKSON.—We learn that Professor Jackson, who so long and very ably filled the chair of Physiology in the University of Pennsylvania, intends at once to tender his resignation. Dr. J. was an enthusiatic, highly interesting, and brilliant lecturer, and inspired his hearers to a great extent with his own zeal for physiological investigations. We wish him in his retirement the repose he is so well entitled to, with health, long life, and every comfort.—*Medical News and Library.*

---

CONTAGION OF SECONDARY SYPHILIS.—Among the victims of secondary syphilis, writes M. Diday in *Gazette de Lyons*, are the glass-blowers at Giers and Vernasion. The frequency of syphilis among this class of workmen has long been observed, and the fact also that the disease almost always commences in the month. Three individuals are obliged to blow forcibly, one immedately after the other, into a hot iron tube, which they are forced to compress strongly with their lips. Hence, therefore, if in one of the three syphilitic disease of the mouth should exist, its propagation is readily effected. At Lyons we continually meet with cases of syphilis which have been contracted in this way; and occasionally there arise actual epidemics of the disease. M. Diday has presented this state of affairs to the magistrate of the district, and has recommended that a capable physician should be appointed to superintend the *blowers* in these glass establishments, and to prevent any one who has a syphilitic disease of the mouth using the tube alluded to.—*British Medical Journal*, Nov. 29, 1862.

---

THE MARSHALL HALL PHYSIOLOGICAL TEST FOR STRYCHNINE.—It is related of Dr. Marshall Hall, in his biography, that at one time the number of criminal poisoning cases, and the conflicting evidence in regard to the employment of strychnine in some of these set him thinking on the subject. His long series of experiments on the nervous system has rendered him well acquainted with the action of strychnine upon the frog, and "it occurred to him that the extreme susceptibility of this animal to the influence of strychnine would constitute it the most delicate test of its presence, thus substituting a physiological for a chemical test. Aided by Mr. Bullock, of Hanover Street, he performed a series of experiments, by which it was at length satisfactorily demonstrated that a young frog might be rendered tetanic by the *five-thousandth part of a grain* of strychnine!" These experiments were communicated by him in detail to *The Lancet* in 1856. This ingenious suggestion of our great English physiologist has recently been acted upon at North Shields, in the *post mortem* examination of Mrs. Gilhespie, an account of which will be found in our pages. It is stated in *The Times* of November 8th, that "a *drop* of an acidulated solution of the residue from the stomach and duodenum brought in contact with the skin of a young frog produced violent convulsions. Three or four additional drops were applied, with intermissions, and it died at the expiration of half an hour."—*London Lancet.*

---

PERSONAL.—Surgeon Horace Wardner, formerly one of the faculty of the Lind University in this city, who has been in the service since the organization of Col. John McArthur's (12th) Regiment, is spending a ten days' leave of absence with his Chicago friends. The doctor has been, since he left home, in charge of the hospitals at Paducah and Corinth, besides on extended service in the field, and was, until recently, Assistant Medical Director upon Gen. Grant's staff, from which position he has been promoted to Medical Director of the Department of Cairo, with his headquarters at Mound City. He has achieved a fine reputation in the army as a gentleman and an operator. His health has suffered considerably from the climate into which the discharge of his duties called him, but, with his more northern location, it will, doubtless, be restored to him.

---

Medical Secrecy.—The Medical Societies of Paris are at present exercised in regard to the question, whether a physician when consulted with regard to the health of a patient in reference to marriage, should refuse to give any information? The societies of the viii and ix arrondissements have decided as to the obligation of secrecy; while the society of the vii arrondissement have declared that while in general, the above rule is correct, there are also circumstances in which the dictates of conscience are the above law [a higher law]. This last seems to us to be a dangerous decision, and one which might lead to great abuses.

Knowledge gained by a physician in his professional capacity should be deemed sacred, and not to be divulged under any circumstances. It is very questionable whether it be safe to make any exceptions to this rule, and if any be made, they must be extremely rare.—*Medical News and Library.*

---

Curara in Hydrophobia.—The Commission appointed at the Milan Hospital, for the purpose of testing the value of curara as a remedy for hydrophobia, have reported that it has failed to establish its claim as a remedy.—*London Lancet.*

---

Remarkable Instance of the Glancing of a Minie Ball. —Sir: The accompanying case appears to me to be one of unusual interest. If it deserves your attention, you are at liberty to publish it.

Geo. Fowler, Co. F. 50th N. Y., was admitted into hospital, December 19th, with a gun-shot wound received at Fredericksburg, December 13th, the ball entering just behind the left great trochanter, but not emerging. A probe following the track of the ball made an *obtuse* angle of about 115° with the shaft of the femur. A small fragment of bone was found splintered from the great trochanter and extracted.

Jan. 3d.—I discovered tenderness and a point of hardness at upper border of left nates, which was suspected to be the ball, but from its great depth under the muscles, it was impossible to determine. I conferred with two surgeons, who dissented, on the ground that the ball, if there, *must have glanced at an acute angle.* Still, not being able to account for the tender and indurated spot, and the operation unattended with danger, I cut down two inches through the muscles, and came upon the ball, the *curvature* of which corroborated the supposition as to its direction. Yours, etc.,

GEO. F. FRENCH, M.D., A.A. Surg., U.S.A.

*American Medical Times.*

---

STATISTICS OF THE GLOBE.—The following curious facts are stated by the *Abeille Médicale:*—The earth is inhabited by about 1,288,000,000 of inhabitants, viz.: 369,000,000 of the Caucasian race, 552,000,000 of the Mongolian race, 196,000,000 of the Ethiopian, 1,000,000 of the American Indian, and 200,000,000 of the Malay races. All these respectively speak 3064 languages, and profess 1000 different religions. The amount of deaths per annum is 333,333,333, or 91,954 per day, 3730 per hour, 60 per minute, or 1 per second; so that at every pulsation of our hearts, a human being dies. This loss is compensated by an equal number of births. The average duration of life throughout the globe is 33 years. One-fourth of its population dies before the seventh year, and one-half before the seventeenth. Out of 10,000 persons, only one reaches his hundredth year, only one in 500 his eighteenth, and only one in 100 his sixty-fifth. Married people live longer than unmarried ones, and a tall man is likely to live longer than a short one. Until the fiftieth year, women have a better chance of life than men, but beyond that period the chances are equal. Sixty-five persons out of one thousand marry. The months of June and December are those in which marriages are most frequent. Childern born in spring are generally stronger than those born in other seasons. Births and deaths chiefly occur at night. The number of men able to bear arms is but an eighth of the population. The nature of the profession exercises a great influence on longevity: thus out of 100 of each of the following professions the number of those who attain their seventieth year is—among clergymen, 42; agriculturists, 40; trades and manufacturers, 33; soldiers, 32; clerks, 32; lawyers, 29; artists, 28; professors, 27; and physicians, 24; so that those who study the art of prolonging the lives of others are most liable to die early, probably on account of the effluvia to which they are constantly exposed. There are in the world 335,000,000 of Christians, 5,000,000 of Jews, 600,000,000 professing some of the Asiatic religions, 160,000,000 of Mahometans, and 200,000,000 of Pagans. Of the Christians, 170,000,000 profess the Roman Catholic, 76,000,000 the Greek and 80,000,000 the Protestant creeds.—*Boston Med. and Surg. Journal.*

